# Clinical correlates and prognostic impact of binge-eating symptoms in major depressive disorder

**DOI:** 10.1097/YIC.0000000000000422

**Published:** 2022-07-12

**Authors:** Paolo Olgiati, Giuseppe Fanelli, Anna Rita Atti, Diana De Ronchi, Alessandro Serretti

**Affiliations:** a Department of Biomedical and Neuromotor Sciences, University of Bologna, Bologna, Italy; b Department of Human Genetics, Radboud University Medical Center, Donders Institute for Brain, Cognition and Behaviour, Nijmegen, The Netherlands

**Keywords:** antidepressant response, binge, eating, bipolar spectrum, major depression, mixed features, suicide

## Abstract

Binge-eating (BE) symptoms are relatively common in major depressive disorder (MDD), but their prognostic role is not fully understood. This study compared two groups of patients with MDD experiencing or not BE symptoms to ascertain differences in terms of clinical manifestations, presence of bipolar features, and antidepressant treatment outcomes. The study involved 482 outpatients collected within the Combining Medications to Enhance Depression Outcomes (CO-MED) trial, who were assessed with scales for depressive and hypomanic symptomatology, suicidality, comorbid mental disorders, and childhood traumas. BE symptoms were reported in 95 patients (20%). Patients with MDD experiencing BE symptoms were characterized by higher scores of negative self-outlook (*P* = 0.0018), negative outlook of future (*P* = 0.0014), irritability (*P* = 0.0043), comorbid anxiety disorders (generalized anxiety disorder: *P* = 0.0006; panic disorder: *P* < 0.0001; social phobia: *P* < 0.0001), obsessive-compulsive disorder (*P* = 0.0053), hypomanic symptoms (increased talkativeness: *P* = 0.0029; reduced need for sleep: *P* = 0.0171), and suicidality (suicidal propensity: *P* = 0.0013; suicidal risk: *P* = 0.0148; lifetime suicidal behavior: *P* = 0.0052). BE symptoms (OR = 2.02; 95% CI = 1.06–3.84) and depression severity (OR = 1.04; 95% CI = 1.00–1.08) were independently associated with lifetime attempted suicide. The presence of BE symptoms might indicate higher severity of depressive disorder. Suicidal risk is a major issue in these patients, whereas the association between BE and bipolar features needs further research.

## Introduction

Symptoms of binge-eating (BE), defined as the intake of a large amount of food in a short period of time often preceded or followed by negative affect (Wolfe *et al.*, 2009), are present in all eating disorders (EDs) and also in subthreshold forms (Johnson *et al.*, 2021). The prevalence of any BE symptom, which is about 4.5% in the general population (Hudson *et al.*, 2007), might be considerably higher among individuals with mood disorders. Individuals with severe obesity who have undergone bariatric surgery represent a well-established cohort in which BE symptoms have been studied. In this group, studies have reported rates of binge-eating disorder (BED) exceeding 30%, whereas bipolar disorder (BD) and major depressive disorders (MDD) were each diagnosable in at least 15% of individuals; mood disorders have been associated with a more than two-fold increased risk of EDs (Barbuti *et al.*, 2021). According to a recent meta-analysis, in an overall sample of more than 15 000 patients with BD, the prevalence rates of bulimia nervosa and BED were 7.4 and 12.5%, respectively (Fornaro *et al.*, 2021). The prevalence of eating symptoms and disorders in patients with a primary diagnosis of MDD was the focus of two recent studies. One study, which included more than 122 000 adolescent inpatients, identified 1675 cases (1.4%) with a comorbid ED (Patel *et al.*, 2021). The other, including more than 800 adults with MDD from a large epidemiological study carried out in South Korea, showed BE symptoms in 17.4% of the sample (Baek *et al.*, 2018). Both studies explored several clinical features associated with ED comorbidity but, among these, no possible marker of the bipolar spectrum. This may be regarded as a limitation in view of the established links between ED and BD. Moreover, prior studies have not investigated the impact of ED comorbidity on antidepressant response.

In the current study, we analyzed a sample of outpatients with MDD and compared subjects who endorsed BE symptoms and their counterpart who did not. Our aims were: (a) to identify the clinical variables associated with BE symptoms; (b) to ascertain whether a number of bipolar spectrum validators were correlated with BE symptoms; (c) to ascertain whether BE symptoms could affect antidepressant treatment outcomes.

## Methods

### Sample

This study included participants in the Combining Medications to Enhance Depression Outcomes (CO-MED) trial (Rush *et al.*, 2011). Their eligibility criteria were age between 18 and 75 years old, a primary Diagnostic and Statistical Manual of mental disorders (DSM)-IV-based diagnosis of non-psychotic MDD, and a 17-item Hamilton Depression rating scale (HAM-D17) score of at least 16. Conversely, any bipolar or psychotic disorder was reason for exclusion, along with the need for hospital treatment.

The CO-MED trial was conducted according to the Principles of the Helsinki Declaration, and its protocol was approved by the ethical committees at each recruitment site. All subjects who met the selection criteria were included in the CO-MED trial after obtaining their written informed consent. This research group certifies that data collected from the CO-MED trial were exclusively used for scientific investigation, and, before access was granted, the objectives of our investigation were clearly reported in the request form (Olgiati and Serretti, 2022).

### Treatments and data collection

The CO-MED trial was characterized by a single-blind placebo-controlled design and three treatment arms: (a) escitalopram plus placebo; (b) bupropion sustained-release plus escitalopram; (c) venlafaxine extended-release plus mirtazapine. The trial included a short-term (12 weeks) treatment followed by a continuation phase (weeks 12–28) (Rush *et al.*, 2011).

Research data were collected by a variety of assessment tools as reported in our previous publications (Olgiati and Serretti, 2022): (a) the socio-demographic form including age, sex, ethnic group, education, and monthly income; (b) the Mini International Neuropsychiatric Interview (MINI) (Sheehan *et al.*, 1998), which was used to assess the chronic or recurrent course of MDD, the number of past depressive episodes, age at onset of first depressive episode, as well as to ascertain the lifetime occurrence of subthreshold hypomanic episodes; (c) a battery of scales for depressive episodes assessment, including the 30-item Inventory of Depressive Symptomatology-Clinician Rating (IDS-C_30_) (Corruble *et al.*, 1999) and the 16-item Quick Inventory of Depressive Symptomatology (QIDS-C_16_) (Rush *et al.*, 2003), the Concise Associated Symptoms Tracking (CAST) for irritability (Trivedi *et al.*, 2011a), the Concise Health Risk Tracking (CHRT) (Trivedi *et al.*, 2011b) for suicidality, the Altman Self-Rating Mania (ASRM) scale (Altman, 1998) for hypomanic symptoms occurring within depressive episodes, and the Work and Social Adjustment Scale (Mundt *et al.*, 2002) to ascertain functional impairment; (d) the Psychiatric Diagnostic Screening Questionnaire (PDSQ) (Zimmerman and Mattia, 1999) to assess comorbid psychiatric disorders, and (e) two questionnaires that were specifically developed for the CO-MED trial in order to investigate lifetime suicidal behavior (Serretti *et al.*, 2021) and experiences of neglect and abuse during childhood (Olgiati and Serretti, 2022).

### Binge-eating symptoms

BE symptoms were investigated by means of the PDSQ, subscale for EDs (PDSQ-ED), which was composed by 10 items and characterized by an assessment time of 2 years (Table 1). A score of 7 was selected as a clinical significance threshold. This cut-off score was associated with high (>0.85) sensitivity and specificity values (Zimmerman and Mattia, 2001).

**Table 1:** Binge-eating symptoms as assessed by the Psychiatric Diagnostic Screening Questionnaire, subscale for eating disorders (PDSQ-ED)

*Scoring items*	
During the past 2 years	
44. Did you often go on eating binges (eating a very large amount of food very quickly in a short period of time)?	1
45. Did you often feel you could not control how much you were eating during an eating binge?	1
46. Did you go on eating binges during which you ate so much that you felt uncomfortably full?	1
47. Did you go on eating binges during which you ate a large amount of food even when you didn’t feel hungry?	1
48. Did you eat alone during an eating binge because you were embarrassed by how much you were eating?	1
49. Did you go on eating binge and then feel disgusted with yourself after overeating?	1
50. Were you very upset with yourself because you were going on eating binges?	1
51. To prevent gaining weight from an eating binge did you force yourself to vomit or use laxatives or water pills?	1
52. To prevent gaining weight from an eating binge did you go on strict diets or exercise excessively?	1
53. Was your weight or the shape of your body one of the most important things that affected your opinion of yourself?	1
**Clinical cut-off score: 7**	

### Statistical analyses

Univariate analyses were performed using Student’s *t*-tests and Chi-square tests for continuous and categorical variables, respectively. A number of clinical variables were compared between patients with (PDSQ-ED *≥* 7) and without BE symptoms (PDSQ-ED *<* 7), including (a) chronic depression: depressive episode lasting for at least six months; (b) depressive symptom profile (IDS-C_30_): (i) negative self-outlook, (ii) negative outlook of future, (iii) loss of pleasure, (iv) anxious mood, (v) interpersonal sensitivity, (vi) poor concentration, (vii) sleep onset insomnia, (viii) middle (ix) nocturnal insomnia, (x) early awakening; (c) irritability (CAST); (d) bipolar spectrum validators: (i) mixed depression, defined as a major depressive episode with three or more concurrent hypomanic symptoms (ASRM items: cheerfulness, self-confidence, reduced need for sleep, talkativeness, goal-oriented hyperactivity); (ii) lifetime subthreshold hypomania: a period of elated or irritable mood with at least two concurrent hypomanic symptoms (MINI interview), which did not fulfil DSM criteria for hypomanic/manic episode (Angst *et al.*, 2003; Serretti, De Ronchi and Olgiati, 2021); (iii) age at onset of first depressive episode (<21 years) (Benazzi, 2009); (iv) major depressive disorder recurrence (episodes/year) (Mazzarini *et al.*, 2018); (e) suicidality: suicidal tendencies were analyzed within depressive episodes by means of CHRT propensity and risk scales, as well as according to a lifetime perspective by a scale ranging between 0 (no suicidal tendency) and 7 (suicide attempt with definite intent to die); (f) comorbid mental disorders, including anxiety disorders, obsessive-compulsive disorder, and alcohol/substance-use disorders (PDSQ); (g) traumatic experiences occurring during childhood: parental neglect, emotional abuse, physical abuse, and sexual abuse; and (h) antidepressant treatment outcomes including response (>50% decrease in QIDS from baseline), remission (QIDS*<*5) rates assessed after six weeks of treatment, and hypomanic switches (ASRM score *>*6) (Altman, 1998) reported after more than 14 days of antidepressant use (Olgiati and Serretti, 2022).

Multivariate analyses were conducted by means of multiple linear and logistic regressions. Statistical software was OpenStat version 8 December 2014 (https://openstat.info/OpenStatMain.htm). Due to the large number of comparisons, the statistical significance threshold was conservatively set at alpha = 0.025 but without a formal correction for multiple testing given the a-priori hypothesis (Amrhein, Greenland and McShane, 2019).

A preliminary power analysis was carried out via G*Power 3 (Faul *et al.*, 2007) to estimate minimum detectable differences between the BE groups, considering a type I error (alpha level) of 0.025 and a type II error (1-power) of 0.20.

## Results

Of 665 participants in the CO-MED trial, 482 were included in the current study. The sample comprised 95 (20%) patients with MDD and BE symptoms (PDSQ-ED ≥ 7) and 387 (80%) controls with MDD but not ED symptoms (PDSQ-ED < 7). Such groups had an adequate power (0.8) to detect small-medium differences, corresponding to more than 0.24 effect sizes (d) (or more than 5% for categorical variables).

### Binge-eating symptoms, demographic features and depression clinical profile

Socio-demographic characteristics and depression symptom profile are reported in Table 2. Demographic features were similarly distributed between the comparison groups. On the contrary, with regard to symptomatology, patients who endorsed BE symptoms were characterized by more severe depression (IDS-C_30_: *t* = 3.053, *P* = 0.0063) and higher scores of negative self-outlook (*t* = 3.496, *P* = 0.0018), negative outlook of future (*t* = 3.202, *P* = 0.0014), and irritability (CAST: *t* = 3.318, *P* = 0.0043) in comparison with their counterpart without BE.

**Table 2:** Socio-demographic and depression characteristics of patients with major depressive disorder experiencing or not binge-eating symptoms

	With BE (*N* = 95)	Without BE (*N* = 387)	*t* or χ^2^	*P*-value
Age	41.91 ± 12.46	43.45 ± 12.45	1.083	0.2792
Sex, *N* males (%)	32 (0.33)	112 (0.29)	0.792	0.3730
Ethnic group, *N* Caucasians (%)	63 (0.66)	258 (0.67)	0.009	0.9990
Education (years)	13.90 ± 2.91	13.68 ± 3.13	0.638	0.5230
Chronic depression, *N* (%)	58 (0.61)	299 (0.51)	3.213	0.0730
IDS-C_30_				
*Total score*	**40.94 ± 7.92**	**38.06 ± 9.33**	**3.053**	**0.0063**
*Negative self-outlook*	**2.16 ± 0.82**	**1.82 ± 0.96**	**3.496**	**0.0018**
*Negative outlook of future*	**1.77 ± 0.76**	**1.46 ± 0.87**	**3.202**	**0.0014**
*Loss of pleasure*	1.59 ± 0.83	1.45 ± 0.91	1.371	0.1742
*Anxious mood*	1.90 ± 0.83	1.83 ± 0.78	0.383	0.7016
*Psychomotor agitation*	0.61 ± 0.59	0.72 ± 0.64	1.531	0.1265
*Slowing*	0.70 ± 0.62	0.74 ± 0.63	0.478	0.6330
*Interpersonal sensitivity*	1.46 ± 1.09	1.41 ± 1.00	0.469	0.6392
*Sleep onset insomnia*	2.06 ± 1.27	1.84 ± 1.28	1.548	0.1224
*Middle nocturnal insomnia*	2.17 ± 1.05	2.07 ± 1.14	0.790	0.4300
*Early awakening*	1.22 ± 1.31	1.23 ± 1.25	0.062	0.9508
*Poor concentration*	1.76 ± 0.73	1.72 ± 0.76	0.487	0.6266
Irritability (CAST)	**0.84 ± 0.37**	**0.70 ± 0.46**	**3.318**	**0.0043**
Work/social impairment	28.67 ± 7.70	26.67 ± 8.98	2.200	0.0467

The mean ± standard deviation is shown for each variable, unless otherwise specified. Significant *P*-values are in bold.

BE, binge-eating symptoms; CAST, concise associated symptoms tracking; IDS-C_30_, 30-item Inventory of Depressive Symptomatology-Clinician Rating.

Negative self-outlook (OR = 1.36, 95% CI = 1.03–1.79), negative outlook of future (OR = 1.41, 95% CI = 1.04–1.91), and irritability (OR = 2.02, 95% CI = 1.09–3.72) were confirmed to predict BE symptoms after controlling for overall depression score (Table 3).

**Table 3:** Clinical predictors of binge-eating symptoms in major depressive disorder

	OR (95% CI)
Negative self-outlook	**1.36 (1.03–1.79**)
Negative outlook of future	**1.41 (1.04–1.91**)
Psychomotor agitation	0.69 (0.49–1.01)
Sleep onset insomnia	1.06 (0.97–1.29)
Poor concentration	0.86 (0.66–1.21)
Irritability	**2.02 (1.09–3.72**)
Work/social impairment	1.01 (0.98–1.04)

Each clinical variable having a *P* < 0.15 in univariate analyses was tested as a possible predictor of BE symptoms by means of multiple logistic regression (MLR) analysis. In all MLR models, the total depression score was controlled for as a potential confounding factor.

Significance threshold: *P* < 0.025. Significant associations are in bold.

CI, confidence interval; OR, odds ratio.

### Comorbidities, childhood traumas and suicidality

Further characteristics of the BE+ group included more comorbid anxiety disorders (generalized anxiety disorder: *t* = 4.096; *P* = 0.0006; panic disorder: *t* = 3.850; *P* < 0.0001; social phobia: *t* = 5.941; *P* < 0.0001) and obsessive-compulsive disorder (*t* = 2.568; *P* = 0.0053), more childhood traumatic experiences (total number of traumas: *t* = 3.284; *P* = 0.0011; child neglect: χ^2^ = 10.151, *P* = 0.0014; emotional abuse: χ^2^ = 7.888, *P* = 0.0050; sexual abuse: χ^2^ = 8.665, *P* = 0.0032), and increased suicidality (CHRT propensity: *t* = 3.231, *P* = 0.0013; CHRT risk: *t* = 2.446, *P* = 0.0148; lifetime suicidal behaviour: *t* = 2.806, *P* = 0.0052) (Table 4).

**Table 4:** Comorbidities, childhood traumas and suicidality of patients with major depressive disorder experiencing or not binge-eating symptoms

	With BE (*N* = 95)	Without BE (*N* = 387)	*t* or χ^2^	*P*-value
*Comorbidities*				
GAD	**7.57 ± 2.48**	**6.32 ± 3.27**	**4.096**	**0.0006**
Panic disorder	**6.38 ± 5.60**	**3.99 ± 4.60**	**3.850**	**<0.0001**
PTSD	4.12 ± 4.25	3.40 ± 4.04	1.536	0.1251
OCD	**1.55 ± 1.99**	**0.98 ± 1.68**	**2.568**	**0.0053**
Social phobia	**7.68 ± 4.96**	**4.84 ± 4.92**	**5.941**	**<0.0001**
Alcohol	0.76 ± 1.46	0.54 ± 1.34	1.408	0.1598
Substance	0.29 ± 0.93	0.20 ± 0.89	0.931	0.3523
*Childhood traumas*				
Total number of traumas	**1.74 ± 1.48**	**1.20 ± 1.41**	**3.284**	**0.0011**
Neglect	**52 (0.55**)	**142 (0.37**)	**10.151**	**0.0014**
Emotional abuse	**54 (0.57**)	**158 (0.41**)	**7.888**	**0.0050**
Physical abuse	25 (0.26)	84 (0.22)	0.090	0.3423
Sexual abuse	**34 (0.36**)	**81 (0.21**)	**8.665**	**0.0032**
*Suicidality*				
CHRT propensity	**18.62 ± 7.43**	**15.359 ± 8.38**	**3.231**	**0.0013**
CHRT risk	**2.65 ± 2.68**	**1.93 ± 2.97**	**2.446**	**0.0148**
Lifetime suicidal behaviour	**2.76 ± 2.53**	**2.01 ± 2.27**	**2.806**	**0.0052**

Significant *P*-values are in bold.

BE, binge-eating symptoms; CHRT, Concise Health Risk Tracking; GAD, generalized anxiety disorder; IDS-C_30_, 30-item Inventory of Depressive Symptomatology - Clinician Rating; OCD, obsessive-compulsive disorder; PTSD, post-traumatic stress disorder.

Multiple logistic regression (MLR) analysis identified BE symptoms (OR = 2.02; 95% CI = 1.06–3.84) and depression severity (OR = 1.04; 95% CI = 1.00–1.08) as independent predictors of lifetime attempted suicide after controlling for potential confounding factors such as comorbid anxiety disorders, negative outlook of future, and irritability (see the Discussion section). Conversely, if childhood traumatic experiences were included in the MLR model, attempted suicide was predicted by depression severity (OR = 1.04; 95% CI = 1.01–1.07) and sexual abuse (OR = 3.00; 95% CI = 1.57–5.76), whereas the contribution of BE symptoms became nonsignificant (OR = 1.80; 95% CI = 0.96–1.37) (Table 5).

**Table 5:** Predictors of lifetime attempted suicide

	OR (95% CI)
*Exclusion of childhood traumatic experiences*	
Depression severity (total IDS-C_30_)	**1.04 (1.00–1.08**)
Panic disorder	0.99 (0.91–1.07)
GAD	1.02 (0.88–1.16)
PTSD	0.99 (0.92–1.08)
OCD	1.11 (0.94–1.32)
Social phobia	0.98 (0.92–1.06)
BE	**2.02 (1.06–3.84**)
Negative outlook of future	1.08 (0.73–1.60)
Irritability	1.01 (0.46–1.19)
*Inclusion of childhood traumatic experiences*	
Depression severity (total IDS-C_30_)	**1.04 (1.01–1.07**)
BE	1.80 (0.96–1.37)
Neglect	1.13 (0.46–2.78)
Emotional abuse	0.86 (0.33–2.24)
Physical abuse	0.92 (0.41–2.05)
Sexual abuse	**3.00 (1.57–5.76**)

Multiple logistic regression analyses. Significance threshold: *P* < 0.025. Significant associations are in bold.

BE, binge-eating symptoms; CI, confidence interval; CHRT, Concise Health Risk Tracking; GAD, generalized anxiety disorder; IDS-C_30_, 30-item Inventory of Depressive Symptomatology - Clinician Rating; OCD, obsessive-compulsive disorder; OR, odds ratio; PTSD, post-traumatic stress disorder.

### Bipolar features and antidepressant treatment outcomes

The distribution of bipolar validators between the BE+ and BE− groups is reported in Table 6. Patients who experienced BE symptoms showed higher levels of talkativeness (*t* = 2.707; *P* = 0.0029) and reduced need for sleep (*t* = 2.102; *P* = 0.0171) than their counterpart without BE symptoms. In addition, they had more goal-oriented hyperactivity (*t* = 1.554; *P* = 0.0441) and younger age of onset (*t* = 1.890; *P* = 0.0594), although such differences did not reach statistical significance.

**Table 6:** Bipolar features and antidepressant treatment outcomes in patients with major depressive disorder experiencing or not binge-eating symptoms

	With BE (*N* = 95)	Without BE (*N* = 387)	*t* or χ^2^	*P*-value
Age of onset	21.01 ± 12.42	23.93 ± 13.71	1.890	0.0594
Recurrence (episodes/year)	0.38 ± 0.76	0.38 ± 0.78	0.025	0.9801
DMX	17 (0.18)	48 (0.12)	1.972	0.1600
SH	11 (0.12)	37 (0.09)	0.346	0.5560
*Hypomanic symptoms (ASRM*)				
Cheerfulness	0.33 ± 0.64	0.22 ± 0.58	1.610	0.1081
Self-confidence	0.25 ± 0.60	0.26 ± 0.65	0.115	0.9089
Talkativeness	**0.63 ± 0.97**	**0.34 ± 0.80**	**2.707**	**0.0029**
Reduced need for sleep	**0.71 ± 1.19**	**0.43 ± 0.95**	**2.102**	**0.0171**
Goal-oriented hyperactivity	0.29 ± 0.84	0.16 ± 0.53	1.554	0.0441
*Antidepressant treatment outcomes (n = 395; 85 with BE*)				
Response	41 (0.47)	142 (0.45)	0.158	0.6909
Remission	24 (0.28)	93 (0.30)	0.090	0.7644
Hypomanic switches (*n* = 424; 82 with BE)	42 (0.51)	162 (0.47)	0.422	0.5161

Significant differences are in bold.

BE, binge-eating symptoms; DMX, depressive mixed state: a major depressive episode with more than three hypomanic symptoms; SH, subthreshold hypomania: a period of elated or irritable mood with more than two hypomanic symptoms, which does not fulfil DSM criteria for hypomanic/manic episode; ASRM, Altman Self-Rating Mania Scale; Hypomanic switch: ASRM score *>* 6 not present at baseline and occurring after at least 14 days of antidepressant use.

Similar differences in hypomanic symptoms were reported after controlling for depression severity, anxious mood, and insomnia (talkativeness: OR = 1.44; 95% CI = 1.14–1.83; reduced need for sleep: OR = 1.23; 95% CI = 1.01–1.51; goal-oriented hyperactivity: OR = 1.43; 95% CI = 1.03–1.97) (Table 7).

**Table 7:** Hypomanic symptoms independently associated with binge-eating symptoms in major depressive disorder

	X^2^	*P*-value	OR (95% CI)
Talkativeness	**19.09**	**0.0008**	**1.44 (1.14–1.83**)
Reduced need for sleep	**14.11**	**0.0069**	**1.23 (1.01–1.51**)
Goal-oriented hyperactivity	**14.58**	**0.0057**	**1.43 (1.03–1.97**)

A multiple logistic regression (MLR) analysis was performed for each hypomanic symptom separately. In each MLR model, the presence of binge-eating symptoms was the dependent variable, while depression severity, anxious mood, and insomnia were treated as confounding variables.

Significance threshold: *P* < 0.025. Significant associations are in bold.

CI, confidence interval; OR, odds ratio.

Antidepressant treatment outcomes were analyzed in 395 patients who completed a 6-week follow-up. No differences were reported between the BE groups with respect to all outcome parameters (response: χ^2^ = 0.158, *P* = 0.6909; remission: χ^2^ = 0.090, *P* = 0.764). Hypomanic switches were assessed in 424 patients whose baseline ASRM was lower than 6 and who remained in treatment for at least 14 days. No significant association was reported between BE symptoms and hypomanic switches (χ^2^ = 0.422; *P* = 0.5161) (Table 6).

## Discussion

In our study, one in five outpatients with MDD experienced BE symptoms. This proportion was similar to the 17% reported in the Korean Epidemiologic Catchment Area (KECA) study, in which, however, only 8% of patients experienced BE symptoms frequently (Baek *et al.*, 2018). Unlike clinical variables that are discussed below, demographic characteristics were not found to affect the distribution of BE symptoms. In particular, their distribution was not different in the subgroups of men and women. According to recent meta-analytic data, EDs are approximately three times more prevalent in women than in men, but while in anorexia nervosa women are 15 times more represented, in bulimia nervosa and BED women-to-men ratio decreases to 3:1 and 2:1, respectively (van Eeden *et al.*, 2021; Qian *et al.*, 2022). There are also studies in which sex-related differences are less clear-cut. For example, a retrospective analysis of adolescent bariatric surgery candidates revealed that, in about one-third of the sample endorsing both BE and compensatory symptoms, there were negligible sex differences (Cheng *et al.*, 2021). In the already-mentioned Korean study (Baek *et al.*, 2018), 36 out of 142 patients with BE (25%) were men compared with 22% in the overall sample. In the last few years, research has shed more light on the role of sex in EDs, pointing to differences in stress hormones and the ghrelin system (Yamada, 2021).

By analyzing the prognostic role of BE symptoms, they emerged as a strong risk factor for suicidality. In fact, they were directly correlated with lifetime suicide attempts and suicidal ideation during depressive episodes, and, notably, such correlations were independent of depression severity. These findings are largely consistent with prior literature, showing that suicide attempt risk was increased in clinically diagnosed EDs and related symptoms (Mandelli *et al.*, 2019; Smith *et al.*, 2019). In patients with BD, ED psychopathology has also been associated with mood instability and suicidality (McDonald *et al.*, 2019). Finally, more straightforwardly related to current results, the KECA study reported an association between BE symptoms and suicide attempts after controlling for age, sex, and comorbidities (Baek *et al.*, 2018).

Little is known about psychopathological connections between BE symptoms and suicidality. We tested the hypotheses of an indirect connection mediated by depressive symptoms that are known to increase suicide-related outcomes, such as hopelessness (Wolfe *et al.*, 2017; Ribeiro *et al.*, 2018) and irritability (Orri *et al.*, 2018; Jha *et al.*, 2020; Serretti *et al.*, 2021), or by psychiatric comorbidities. Both these symptoms and anxiety or obsessive-compulsive disorder comorbidities were reported at higher levels in the subgroup of patients with BE symptoms. Nevertheless, the association between BE symtoms and attempted suicide remained significant after controlling for these potential confounding factors, suggesting that the prosuicidal influence of BE symptoms might be independent. In addition, we tested the hypothesis that traumatic experiences occurring in childhood, which are demonstrated to facilitate suicidal behavior in adults (Angelakis *et al*., 2019), might act as interplaying variables between BE symptoms and lifetime suicidality. In line with a prior study (Baek *et al.*, 2018), we observed that the association between BE symptoms and attempted suicide became nonsignificant after controlling for childhood traumas, whereas sexual abuse was confirmed to be a prominent suicidal risk factor. BE symptoms and suicidal behavior might share underlying affect dysregulation (Lavender *et al.*, 2015) and impulsivity (Giegling *et al.*, 2009; Waxman, 2009). Biologically, BE might cause alterations in tryptophan intake and serotonin levels (Mann and Currier, 2010) or lipid metabolism (Atmaca *et al.*, 2002), which eventually may increase suicide risk.

We also examined the relationship between BE symptoms and bipolar spectrum validators. This aspect, which has not been addressed in prior publications, is important for clinical practice. In fact, individuals with subthreshold bipolar depression are supposed to be at increased risk for inappropriate treatment and complications such as manic switches and rapid cycling (Nusslock and Frank, 2011). Another serious threat for them is suicidality (Akiskal *et al.*, 2005). In the present study, both increased talkativeness and reduced need for sleep, which are two hypomanic symptoms, were associated with BE symptoms, whereas overactivity did not reach statistical significance. Conversely, BE symptoms were not correlated with manic switches or other bipolar validators. These findings did not support a correlation between BE and subthreshold bipolarity. However, since there were few positive associations, further research is warranted.

The impact of BE symptoms on antidepressant treatment outcomes was also explored. Even though patients experiencing BE symptoms had more severe depression at baseline, we were unable to identify a significant association with response or remission. Since it has been demonstrated that initial depression severity does negatively correlate with the likelihood of remitting in a few treatment weeks (Friedman *et al.*, 2012; Falola *et al.*, 2017), our findings might imply that patients with BE symptoms would be more sensitive to antidepressant treatment. There could be a biological explanation regarding a role for serotonergic dysfunction, and possible potentiation with targeted treatments such as pimavanserin could be evaluated (Papakostas *et al.*, 2020). At present, however, the impact of BE symptoms on antidepressant response remains merely a hypothesis that needs to be demonstrated by further studies.

Our study includes strengths and limitations. Strengths are the accurate clinical evaluation of patients in the CO-MED study and the inclusion of variables not analyzed in other similar previous works (i.e., bipolar validators and hypomanic switches). The main limitation of the current post hoc analysis is related to the primary design of the CO-MED study, which was more suitable for a pharmacological trial than specifically for psychopathologic research. Some caveats of PDSQ as a diagnostic tool for nonpsychotic mental disorders, including EDs, were discussed elsewhere (Zimmerman and Chelminski, 2006; Larun *et al.*, 2007). Other caveats involved the assessment of bipolar spectrum validators. Hypomanic symptoms more often reported in mixed depression such as racing thoughts, distractibility, and excessive involvement in risky activities could not be ascertained in BE groups because they are not explored by ASRM items. Moreover, there could be the onset of hypomanic symptoms not investigated by the ASRM scale during antidepressant treatment and, therefore, not identifiable as hypomanic switches. A family history of BD is one of the most useful variables to distinguish bipolar forms (Zimmerman *et al.*, 2013), but it was not available for the CO-MED sample. Affective instability and suicidality are common features of borderline personality disorder, which in turn is often reported as a comorbidity of EDs (Martinussen *et al.*, 2017). However, the CO-MED study did not include instruments to assess personality disorders. Finally, with regard to suicidal behaviour, no information was available about the characteristics of suicide attempts (impulsive or based on plans, use of potentially lethal methods, etc.), their related environmental factors, and symptomatology prior to their occurrence (suicidal ideation, hypomanic symptoms, etc.).

### Conclusion

In conclusion, binge-eating symptoms are present in a significant proportion of patients diagnosed with major depressive disorder, and they play a prominent prognostic role because of their strong association with suicidality. On the other hand, the use of binge-eating symptoms as markers of a potential bipolar diathesis is supported by little clinical evidence, and further research will be needed to further verify their possible usefulness.

## Acknowledgments

We thank the NIMH for having had the possibility to analyze their data on the CO-MED sample. We also thank the authors of previous publications in this dataset, and foremost, we thank the patients and their families who accepted to be enrolled in the study. Data and biomaterials were obtained from the limited access datasets distributed from the NIH. The ClinicalTrials.gov identifier is NCT00590863.

### Conflicts of interest

AS is or has been a consultant/speaker for Abbott, Abbvie, Angelini, AstraZeneca, Clinical Data, Boehringer, Bristol-Myers Squibb, Eli Lilly, GlaxoSmithKline, Innovapharma, Italfarmaco, Janssen, Lundbeck, Naurex, Pfizer, Polifarma, Sanofi, Servier, and Taliaz. The other authors declare no conflicts of interest.
